# Volatiles from *Aquilaria sinensis* damaged by *Heortia vitessoides* larvae deter the conspecific gravid adults and attract its predator *Cantheconidea concinna*

**DOI:** 10.1038/s41598-018-33404-z

**Published:** 2018-10-10

**Authors:** Haili Qiao, Pengfei Lu, Sai Liu, Changqing Xu, Kun Guo, Rong Xu, Jun Chen

**Affiliations:** 1 Institute of Medicinal Plant Development, Chinese Academy of Medical Sciences & Peking Union Medical College, Beijing, 100193 China; 2 Key Laboratory for Silviculture and Conservation of Ministry of Education, Beijing Forestry University, Beijing, 100083 China

## Abstract

The effects of induced plant responses on herbivores are categorised as direct, by reducing herbivore development, or indirect, by affecting the performance of natural enemies. Here, we investigated a tritrophic system, which included the herbivore *Heortia vitessoides*, its host plant *Aquilaria sinensis*, and its predator *Cantheconidea concinna*. Herbivore-damaged *A. sinensis* plants released significantly greater amounts of volatiles than undamaged and mechanically damaged plants, with an obvious temporal trend. One day after initial herbivore damage, *A. sinensis* plants released large amounts of volatile compounds. Volatile compounds release gradually decreased over the next 3 d. The composition and relative concentrations of the electroantennographic detection (EAD)-active compounds, emitted after herbivore damage, varied significantly over the 4-d measurement period. In wind tunnel bioassays, mated *H. vitessoides* females showed a preference for undamaged plants over herbivore and mechanically damaged *A. sinensis* plants. In Y-tube bioassays, *C. concinna* preferred odours from herbivore-damaged plants to those from undamaged plants, especially after the early stages of insect attack. Our results indicate that the herbivore-induced compounds produced in response to attack by *H. vitessoides* larvae on *A. sinensis* plants could be used by both the herbivores themselves and their natural enemies to locate suitable host plants and prey, respectively.

## Introduction

Plants have developed various adaptive and defensive strategies against insect herbivory over the course of their long evolutionary history^1^. Intact, healthy plants normally release various volatile organic compounds (VOCs), which act as important signals for herbivores to locate host plants for oviposition and feeding^2–4^. Plants under herbivore attack respond by emitting much more diverse volatiles in greater quantities compared to healthy, undamaged plants. The diverse volatiles released in response to herbivore attack are termed herbivore induced plant volatiles (HIPVs)^5–7^. HIPVs are more likely to be detected by herbivores, their natural enemies, and neighbouring plants compared to the VOCs released by healthy plants, because of the particular chemical constituents^6^. HIPVs may mediate tritrophic interactions between plants, herbivores, and natural enemies of herbivores, and perhaps even interactions at a fourth trophic level, with hyperparasitoids^8–10^.

*Aquilaria sinensis* (Lour.) Gilg (Thymelaeaceae) is an economically important evergreen tree native to China, which grows mainly in tropical climates, including the provinces of Hainan, Guangdong, Guangxi, Fujian, Yunnan, and Taiwan. *A. sinensis* is the principal source of Chinese agarwood, a resinous *A. sinensis* heartwood formed in response to fungal infection. In China, agarwood is used in religious ceremonies, traditional medicine, and as incense^11^.

*Heortia vitessoides* Moore (Lepidoptera: Crambidae) is the most destructive insect pest of *A. sinensis* throughout the tree’s range in southern China^12^; the hosts of *H. vitessoides* include several species of the genera *Aquilaria* and *Rhus*^13,14^. In China, the larvae feed solely on the leaves of *A. sinensis*^12–14^. Large infestations of these caterpillars have defoliated large areas of forest in southern China and caused significant economic losses. Apart from the heavy use of pesticides, there is no known effective method for controlling this pest.

Su^13^ suggested that the young leaves of *A. sinensis* were the sole emitters of VOCs attractive to *H. vitessoides* females seeking oviposition sites. We previously identified and compared VOCs from young and old *A. sinensis* leaves that potentially attract *H. vitessoides*. We also tested the behavioural responses of *H. vitessoides* to synthetic blends of these VOCs in wind tunnel and field tests, and established a relationship between leaf age preference and host plant recognition in *H. vitessoides*^12^. We found qualitative and quantitative differences between the odour profiles of young and old leaves. Wind tunnel and field tests confirmed that a nine-component mixture based on young leaves (comprised of hexanal, limonene, 2-hexanol, octanal, (*Z*)-3-hexenyl acetate, (*Z*)-3-hexen-1-ol, nonanal, decanal, and 2,6,10-trimethyl-dodecane at a ratio of 2:16:9:4:63:100:13:10:5) attracted significantly more moths than the three component mixture based on old leaves (comprised of nonanal, decanal, and 2,6,10-trimethyl-dodecane in a ratio of 11:14:26). The volatile signals from young *A. sinensis* leaves allowed *H. vitessoides* females to discriminate suitable larval hosts from the background chemical environment, and guided orientation of flights towards these plants for oviposition.

In a more recent study, we found that female adult oviposition on young *A. sinensis* leaves was reduced in response to damage caused by *H. vitessoides* larvae. In other words, female adults preferred to lay eggs on the healthy, intact young leaves. In addition, many natural enemies of *H. vitessoides* larvae, including *Cantheconidea concinna*, are found on herbivore-damaged *A. sinensis* plants^15^. To date many studies have shown that HIPVs can either attract or repel the same or different species of herbivores^16,17^, and even attract their natural enemies^5^. For instance, *Tetranycbus evansi* adults were more attracted to plants attacked by conspecific larvae than to undamaged plants in olfactometer experiments^18^. Caterpillar-induced nocturnal tobacco plant volatiles were found to repel ovipositing conspecific moths^16^. Kappers *et al*.^19^ suggested that HIPVs play a very important role in plant defences against herbivores, both directly and indirectly, as cues that attract predatory and parasitic natural enemies of herbivores.

Here, we hypothesise that HIPVs emitted by *A. sinensis* significantly reduce herbivore oviposition and increase recruitment of their natural enemies, and ask: How do the moth *H. vitessoides* and its predatory enemy *C. concinna* respond to HIPV emissions from *A. sinensis*? We aimed to (1) identify and compare VOCs released by undamaged, mechanically damaged, and herbivore-damaged *A. sinensis* plants; (2) analyse the antennal and behavioural responses of mated *H. vitessoides* females to these volatile compounds; and (3) examine whether HIPVs emitted by *A. sinensis* affect the host-searching behaviour of *C. concinna* on a host-infested plant. In this study, we sought to elucidate the role of HIPVs emitted from herbivore-damaged plants and improve our understanding of how herbivore insects locate hosts and are located by their predators in a tree ecosystem.

## Results

**Figure 1:**
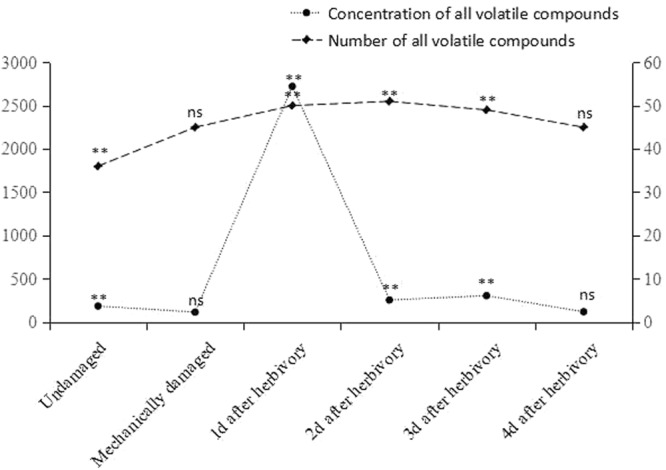
Concentration and number of all volatile compounds detected (±SE) from headspace collections from undamaged, mechanically damaged, and herbivore-damaged *Aquilaria sinensis* plants damaged by leaf-feeding larvae of *Heortia vitessoides* over a 4-d period. *N* = 6 for each treatment group. *P*-values based on one-way ANOVAs conducted at each treatment: ^*^P < 0.05; ^**^P < 0.01; ns = P ≥ 0.05.

The concentrations and compositions of the VOCs, which belonged to eight groups: alcohol, aldehyde, hydrocarbon, ketone, ester, benzenoid, terpenoid, and green leaf volatile, differed significantly among treatments (Table 1). The volatile blends emitted by undamaged, mechanically damaged, and herbivore-damaged *A. sinensis* plants were significantly different, both quantitatively (F = 1315.532, DF = 5, P < 0.001) and to a lesser degree qualitatively (F = 288.600, DF = 5, P < 0.001) (Table 1 and Fig. 1).

**Table 1:** Composition and quantities of volatile compounds in headspace collections from undamaged, mechanically damaged and herbivore-damaged *Aquilaria sinensis* plants.

Compounds	Undamaged^a^	Mechanically damaged^b^	Herbivore-damaged				ANOVA (DF = 5, 30)	Retention time (min)
			1 d^c^	2 d^d^	3 d^e^	4 d^f^		
	C^g^ (R^h^)	C^g^ (R^h^)	C^g^ (R^h^)	C^g^ (R^h^)	C^g^ (R^h^)	C^g^ (R^h^)		
Alcohols								
2,7-dimethyl-2,6-octadien-1-ol	—	—	—	0.08 ± 0.01 (<1)	—	—		6.08
2-ethyl-1,3-hexanediol	—	—	—	0.04 ± 0.01 (<1)	—	—		6.16
2-methyl-5- (1-methylethenyl)-cyclohexanol	—	—	—	—	0.05 ± 0.01b (<1)	0.15 ± 0.01a (<1)	F = 211.89, P < 0.001	6.84
3-methyl-4-heptanol	—	—	0.52 ± 0.09 (<1)	—	—	—		10.16
2-nonen-1-ol	—	—	—	0.14 ± 0.01a (<1)	0.09 ± 0.01b (<1)	0.15 ± 0.01a (<1)	F = 97.79, P < 0.001	10.25
2-decen-1-ol	2.02 ± 0.34b (2.06)	2.42 ± 0.11ab (6.19)	—	1.71 ± 0.05b (1.55)	1.88 ± 0.05b (1.86)	2.45 ± 0.13a (14.39)	F = 4.49, P < 0.01	12.78
1-octen-3-ol	—	—	3.72 ± 0.23 (<1)	—	—	—		12.84
2-hexyl-1-octanol	—	0.11 ± 0.01a (<1)	—	0.08 ± 0.01b (<1)	0.09 ± 0.01b (<1)	0.11 ± 0.01a (<1)	F = 9.52, P<0.001	12.90
2-pentadecyn-1-ol	—	—	1.23 ± 0.08 (<1)	—	—	—		13.21
2-butyl-1-octanol	—	—	—	0.38 ± 0.04a (<1)	0.34 ± 0.02b (<1)	0.42 ± 0.01a (2.44)	F = 12.46, P < 0.05	13.84
1-nonadecanol	—	0.37 ± 0.12 (<1)	—	—	—	—		14.78
2-hexyl-1-decanol	—	—	—	0.28 ± 0.01 (<1)	—	—		14.97
1-eicosanol	—	—	—	—	0.19 ± 0.01a (<1)	0.19 ± 0.01a (1.09)	F = 0.12, P = 0.734	17.03
2-methyl-1-hexadecanol	0.67 ± 0.10c (<1)	0.65 ± 0.01c (1.67)	0.52 ± 0.03d (<1)	0.51 ± 0.04d (<1)	0.85 ± 0.09b (<1)	1.08 ± 0.06a (6.32)	F = 67.43, P < 0.001	24.35
Aldehydes								
2,4-dimethyl-pentanal	—	—	0.72 ± 0.10 (<1)	—	—	—		4.56
heptanal	—	—	—	0.14 ± 0.01b (<1)	—	0.68 ± 0.11a (4.00)	F = 136.93, P < 0.001	6.52
octanal	1.71 ± 0.22c (1.75)	0.93 ± 0.01e (2.38)	—	1.36 ± 0.08d (1.24)	1.88 ± 0.10b (1.22)	2.56 ± 0.11a (15.06)	F = 147.76, P < 0.001	8.86
nonanal	7.43 ± 2.03b (7.65)	5.07 ± 0.14c (12.99)	2.40 ± 0.13d (<1)	6.55 ± 0.23bc (5.96)	4.10 ± 2.74cd (2.84)	10.99 ± 1.37a (64.46)	F = 23.62, P < 0.001	11.39
decanal	9.93 ± 0.46b (10.16)	8.68 ± 0.16c (22.25)	5.94 ± 0.65d (<1)	10.51 ± 0.82b (9.56)	10.05 ± 0.56b (6.57)	16.07 ± 0.34a (94.37)	F = 225.21, P < 0.001	13.95
2-ethylidene-6-methyl-3,5-heptadienal	—	—	1.64 ± 0.05 (<1)	—	—	—		19.06
4- (1-methylethyl)-benzaldehyde	—	0.34 ± 0.12a (<1)	—	0.24 ± 0.02a (<1)	—	—	F = 3.85, P = 0.078	21.35
Hydrocarbons								
dodecane	0.79 ± 0.22a (<1)	0.26 ± 0.01b (<1)	—	—	—	—	F = 35.21, P < 0.001	6.90
tridecane	1.73 ± 0.24a (1.77)	1.28 ± 0.01a (3.28)	—	—	—	—	F = 21.19, P < 0.05	9.20
2-ethenyl-1,1-dimethyl-3-methylene-cyclohexane	—	1.67 ± 0.19d (4.27)	664.99 ± 8.65a (42.92)	110.56 ± 9.30c (100)	153.74 ± 3.69b (100)	17.04 ± 0.62d (100)	F = 146.53, P < 0.001	9.34
2,6,10-trimethyl-dodecane	0.95 ± 0.44a (20.41)	0.30 ± 0.02b (<1)	—	0.20 ± 0.01b (<1)	0.20 ± 0.01b (<1)	0.33 ± 0.01b (1.92)	F = 0.59, P < 0.001	10.56
tetradecane	3.56 ± 0.93a (3.62)	1.88 ± 0.11b (4.85)	1.44 ± 0.11b (<1)	1.60 ± 0.09b (1.45)	1.70 ± 0.04b (1.10)	3.71 ± 0.11a (21.76)	F = 41.99, P < 0.001	11.63
2,6,10,14-tetramethyl-heptadecane	—	—	—	0.77 ± 0.05b (<1)	0.78 ± 0.03b (<1)	1.17 ± 0.11a (6.86)	F = 58.51, P < 0.001	12.58
nonadecane	—	—	—	0.38 ± 0.10 (<1)	—	—		12.95
3-methyl-tridecane	—	—	—	—	0.26 ± 0.01a (<1)	0.34 ± 0.01a (2.00)	F = 367.65, P < 0.001	13.00
pentadecane	3.80 ± 1.02c (3.89)	2.63 ± 0.07d (6.77)	3.50 ± 0.12 cd (<1)	4.61 ± 0.50bc (4.21)	4.94 ± 0.99b (3.18)	6.58 ± 1.08a (38.59)	F = 19.38, P < 0.001	14.06
2,6,10-trimethyl-tetradecane	1.26 ± 0.34b (1.29)	0.47 ± 0.02d (1.22)	1.64 ± 0.10a (<1)	0.80 ± 0.10c (<1)	0.88 ± 0.08c (<1)	0.54 ± 0.11d (3.18)	F = 46.58, P < 0.001	15.38
hexadecane	5.10 ± 0.27d (5.22)	3.90 ± 0.18d (9.99)	6.97 ± 0.43b (<1)	6.16 ± 1.11c (5.66)	7.95 ± 0.70b (5.14)	11.50 ± 1.29a (67.57)	F = 50.285, P < 0.001	16.43
2,6,10-trimethyl-pentadecane	—	—	—	—	2.31 ± 0.11a (4.50)	2.89 ± 0.13a (17.00)	F = 72.34, P < 0.001	16.93
1,2–15,16-diepoxyhexadecane	—	—	—	1.46 ± 0.03b (1.33)	1.53 ± 0.08a (1.00)	1.51 ± 0.06ab (8.85)	F = 2.50, P = 0.116	17.36
6-methyl-octadecane	0.46 ± 0.13b (<1)	0.51 ± 0.19ab (1.23)	0.53 ± 0.05ab (<1)	0.42 ± 0.08b (<1)	0.55 ± 0.11ab (<1)	0.63 ± 0.06a (3.72)	F = 2.48, P < 0.05	17.69
heptadecane	3.24 ± 0.31c (3.32)	2.36 ± 0.15d (6.07)	3.23 ± 0.04c (<1)	4.58 ± 0.39b (4.19)	4.98 ± 0.36b (3.23)	6.09 ± 1.26a (35.77)	F = 34.09, P<0.001	18.71
octadecane	1.21 ± 0.07e (1.24)	0.78 ± 0.01f (2.00)	2.13 ± 0.07a (<1)	1.38 ± 0.11d (1.25)	2.01 ± 0.12b (1.32)	1.70 ± 0.07c (10.00)	F = 220.30, P<0.001	20.89
heptacosane	2.15 ± 0.29d (2.20)	2.15 ± 0.09d (5.53)	2.85 ± 0.06c (<1)	3.82 ± 0.62b (3.48)	5.06 ± 0.53a (3.25)	—	F = 55.38, P<0.001	28.76
heneicosane	4.19 ± 1.14e (4.27)	5.20 ± 0.12d (13.33)	6.32 ± 0.54c (<1)	7.96 ± 0.49b (7.26)	9.77 ± 0.48a (6.38)	1.61 ± 0.09f (9.46)	F = 141.50, P<0.001	30.55
octacosane	8.99 ± 0.79d (9.21)	10.15 ± 0.12c (26.05)	10.12 ± 0.97c (<1)	13.16 ± 0.69b (11.98)	16.77 ± 0.57a (10.86)	2.99 ± 0.12e (17.56)	F = 326.96, P<0.001	35.49
Ketones								
3-eicosanone	—	—	—	0.44 ± 0.07a (<1)	0.22 ± 0.25b (<1)	0.24 ± 0.01b (1.40)	F = 3.84, P<0.05	5.85
6-methyl-5-hepten-2-one	3.51 ± 0.94b (3.62)	3.10 ± 0.17b (7.92)	0.75 ± 0.17c (<1)	3.77 ± 1.32b (3.46)	3.37 ± 0.13b (2.20)	5.28 ± 0.45a (31.03)	F = 26.66, P<0.001	10.00
2-methyl-5- (1-methylethenyl)-cyclohexanone	1.22 ± 0.35a (1.24)	0.43 ± 0.01b (1.10)	—	—	—	—	F = 30.33, P<0.001	14.31
Esters								
butyl propanoate	0.40 ± 0.08b (<1)	—	3.70 ± 0.42a (<1)	0.50 ± 0.09b (<1)	0.39 ± 0.06b (<1)	—	F = 330.34, P<0.001	5.65
butyl isobutyrater	—	—	0.15 ± 0.05 (<1)	—	—	—		5.79
butyl acrylate	0.67 ± 0.12bc (<1)	0.16 ± 0.02c (<1)	8.85 ± 0.15a (<)	0.57 ± 0.24bc (<1)	0.93 ± 0.04b (<1)	—	F = 293.82, P < 0.001	6.31
heptyl butanoate	—	—	6.14 ± 0.77a (<1)	—	0.41 ± 0.62b (<1)	0.07 ± 0.01b (<1)	F = 214.37, P<0.001	7.18
butyl butanoate	4.65 ± 0.66b (4.75)	1.31 ± 0.03b (3.38)	16.72 ± 0.95a (1.08)	2.31 ± 0.18b (2.10)	2.99 ± 0.04b (1.95)	—	F = 7.63, P<0.001	7.28
hexyl acetate	—	—	2.83 ± 0.05 (<1)	—	—	—		8.55
ethenyl octadecanoate	0.24 ± 0.04a (<1)	0.12 ± 0.01b (<1)	—	—	0.23 ± 0.01a (<1)	0.25 ± 0.01a (1.46)	F = 58.03, P<0.001	14.68
methyl benzoate	—	—	13.28 ± 0.78a (<1)	1.73 ± 0.07b (1.58)	0.34 ± 0.01c (<1)	—	F = 1467.20, P<0.001	16.73
7-methyl-z-tetradecen-1-ol acetate	—	0.38 ± 0.02 (<1)	—	—	—	—		19.91
geranyl isovalerate	0.43 ± 0.16a (<1)	—	0.64 ± 0.08a (<1)	—	—	—	F = 8.32, P<0.05	24.54
dibutyl glutarate	0.37 ± 0.08c (<1)	0.42 ± 0.01c (1.07)	0.54 ± 0.09b (<1)	0.56 ± 0.04b (<1)	0.62 ± 0.05b (<1)	1.12 ± 0.09a (6.60)	F = 91.91, P<0.001	25.10
methyl hexadecanoate	0.19 ± 0.01b (<1)	0.36 ± 0.20a (<1)	—	—	—	0.36 ± 0.02a (2.11)	F = 4.35, P<0.05	29.04
ethyl hexadecanoate	—	0.30 ± 0.01c (<1)	0.37 ± 0.24c (<1)	0.51 ± 0.05b (<1)	0.85 ± 0.09a (<1)	0.15 ± 0.01d (<1)	F = 29.99, P<0.001	29.73
Benzenoids								
1,3,5-trimethyl-benzene	—	—	—	—	—	0.15 ± 0.01 (<1)	8.64	
1-ethenyl-4-ethyl-benzene	—	—	—	1.31 ± 0.07b (1.19)	0.68 ± 0.03c (<1)	2.03 ± 0.12a (11.93)	F = 418.14, P<0.001	12.64
methyl salicylate	0.73 ± 0.07b (<1)	—	3.54 ± 0.15a (<1)	—	—	—	F = 421.473, P<0.001	20.13
benzothiazole	0.70 ± 0.06c (<1)	0.51 ± 0.01c (1.30)	0.73 ± 0.07c (<1)	1.30 ± 0.03a (1.18)	1.33 ± 0.58a (<1)	0.75 ± 0.07b (4.42)	F = 32.26, P<0.001	23.87
(Z)-3-hexen-1-ol, benzoate	—	—	0.96 ± 0.09 (<1)	—	—	—		27.24
Terpenoids								
β-myrcene	—	—	9.11 ± 0.79 (<1)	—	—	—		6.12
3-carene	—	—	—	—	—	0.19 ± 0.01 (1.11)	7.04	
eucalyptol	—	—	—	0.20 ± 0.01a (<1)	—	0.22 ± 0.02a (1.31)	F = 13.24, P<0.05	7.12
(E)-β-ocimene	3.60 ± 0.89b (3.67)	1.49 ± 0.09c (3.79)	76.80 ± 2.90a (4.95)	1.42 ± 0.08c (1.29)	0.49 ± 0.14c (<1)	—	F = 64.121, P<0.001	7.61
(Z)-β-ocimene	97.81 ± 4.41b (100)	38.98 ± 1.73c (100)	1549.73 ± 27.24a (100)	42.98 ± 3.89c (39.04)	23.27 ± 0.70d (15.19)	3.91 ± 0.17e (23.02)	F = 228.340, P<0.001	8.00
linalool	—	1.08 ± 0.18c (2.70)	9.54 ± 0.83a (<1)	1.40 ± 0.10bc (1.27)	1.88 ± 0.08b (1.22)	—	F = 533.58, P<0.001	15.14
(+)-longifolene	—	3.15 ± 0.11 (8.07)	—	—	—	—		15.60
globulol	—	—	—	—	—	3.76 ± 0.11 (22.10)	15.65	
epiglobulol	—	0.32 ± 0.01e (<1)	1.44 ± 0.04c (<1)	0.79 ± 0.08d (<1)	6.85 ± 0.56a (4.49)	2.42 ± 0.12b (14.22)	F = 613.18, P<0.001	16.12
caryophyllene	—	0.67 ± 0.11b (1.67)	5.71 ± 1.11a (<1)	—	—	—	F = 122.60, P<0.001	16.28
L- (−)-menthol	2.42 ± 0.10a (2.48)	1.60 ± 0.07b (4.10)	1.18 ± 0.12c (<1)	—	—	—	F = 235.60, P<0.001	17.30
α-farnesene	1.58 ± 0.22d (1.61)	1.19 ± 0.02d (3.07)	99.23 ± 4.21a (6.41)	5.02 ± 0.94c (4.54)	19.26 ± 0.54b (12.55)	1.18 ± 0.13d (6.92)	F = 253.001, P<0.001	19.70
neocurdione	0.87 ± 0.17b (<1)	1.02 ± 0.02a (2.60)	0.63 ± 0.09c (<1)	1.08 ± 0.04a (<1)	1.02 ± 0.04a (<1)	0.56 ± 0.08c (3.28)	F = 37.06, P<0.001	26.85
cedrol	0.66 ± 0.13c (<1)	0.94 ± 0.24b (2.33)	0.56 ± 0.07c (<1)	0.99 ± 0.10b (<1)	1.22 ± 0.10a (<1)	1.21 ± 0.07a (7.12)	F = 26.83, P<0.001	27.17
Green leaf volatiles								
2-ethyl-1-hexanol	4.48 ± 0.99a (4.57)	0.80 ± 0.01c (2.05)	2.16 ± 0.17b (<1)	—	—	—	F = 61.59, P<0.001	13.78
(Z)-3-hexen-1-ol	—	0.71 ± 0.01d (1.81)	24.43 ± 1.09a (1.58)	5.02 ± 0.69b (4.56)	2.98 ± 0.19c (1.93)	0.83 ± 0.08d (4.87)	F = 522.612, P<0.001	11.19
3-hexanol	—	—	4.82 ± 0.41 (<1)	—	—	—		6.79
(E)-2-hexen-1-ol	—	—	1.69 ± 0.12a (<1)	0.43 ± 0.04b (<1)	0.23 ± 0.01c (<1)	—	F = 640.41, P<0.001	11.73
1-hexanol	—	—	—	0.54 ± 0.05a (<1)	0.98 ± 0.14a (<1)	—	F = 52.55, P<0.001	10.46
2-hexenal	—	—	—	0.11 ± 0.01 (<1)	—	—		7.21
3-hexanone	—	—	0.58 ± 0.13 (<1)	—	—	—		4.13
(*Z*)-3-hexenyl acetate	—	2.53 ± 0.29b (6.51)	144.70 ± 9.40a (9.34)	1.12 ± 0.06b (1.02)	0.59 ± 0.15b (<1)	—	F = 57.250, P<0.001	9.53
2-hexen-1-ol, acetate	—	—	9.88 ± 0.67 (<1)	—	—	—		9.96

^a^Undamaged plants. Plants had no any insect feeding and mechanically damage (N = 6).

^b^Mechanically damaged plants. The leaves of plants were cut with a razor blade (N = 6).

^c^Plants that were fed upon by late-third to fourth-instar larvae of *Heortia vitessoides* at 1 d after damage and removing caterpillars (N = 6).

^d^Plants that were fed upon by late-third to fourth-instar larvae of *Heortia vitessoides* at 2 d after damage and removing caterpillars (N = 6).

^e^Plants that were fed upon by late-third to fourth-instar larvae of *Heortia vitessoides* at 3 d after damage and removing caterpillars (N = 6).

^f^Plants that were fed upon by late-third to fourth-instar larvae of *Heortia vitessoides* at 4 d after damage and removing caterpillars (N = 6).

^g^The average concentration of each compound collected from 100 g of undamaged, 100 g of mechanically damaged and 100 g of herbivore-damaged *Aquilaria sinensis* plants of different treatment in ng/g.

^h^Amounts relative to the most abundant compound (set at a value of 100).

### Identification of plant volatiles quantitatively

**Figure 2:**
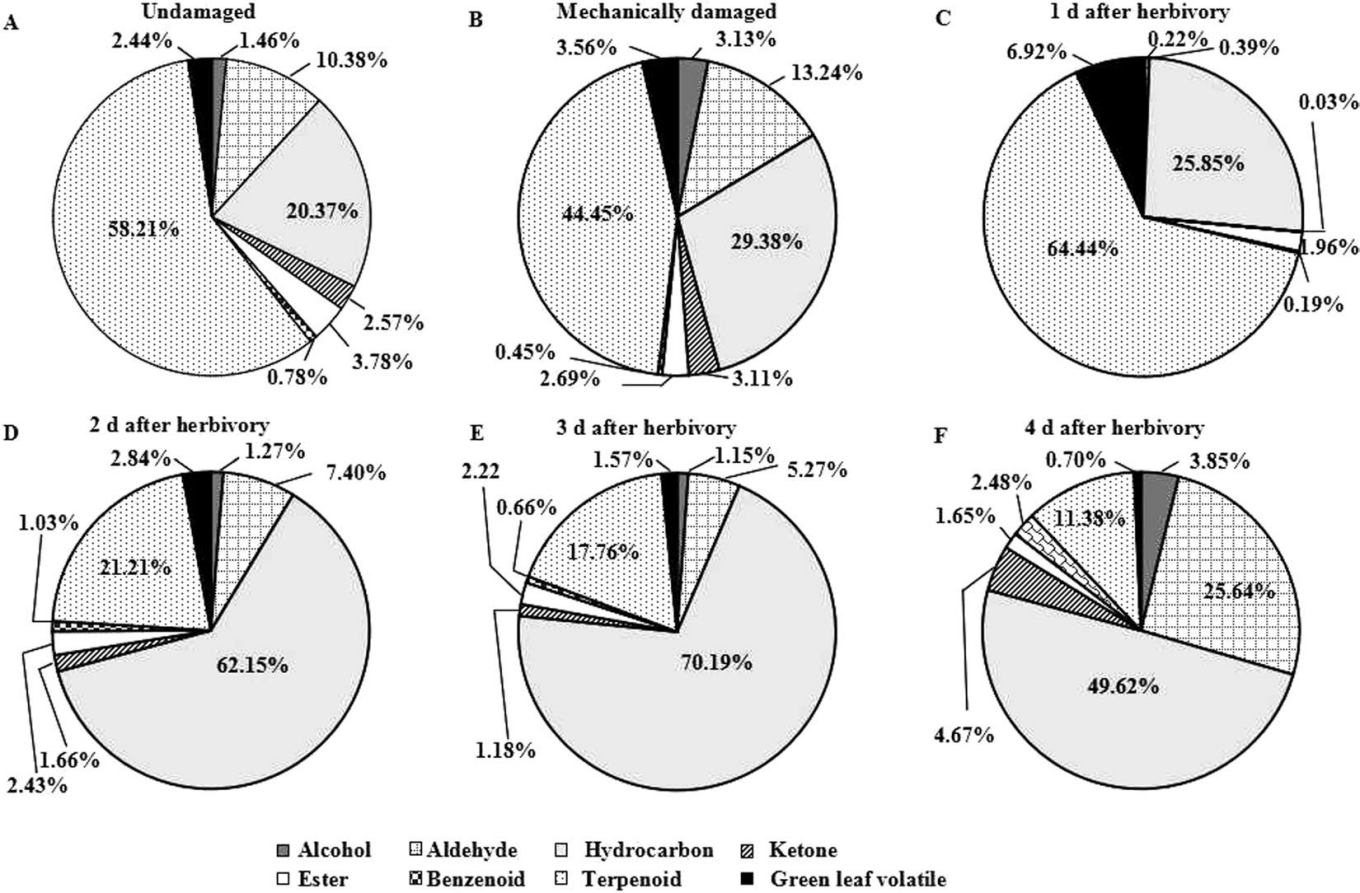
Quantitative proportions of major classes of volatile organic compounds in the headspace collected from undamaged, mechanically damaged, and herbivore-damaged *Aquilaria sinensis* plants damaged by leaf-feeding larvae of *Heortia vitessoides* over a 4-d period.

The total absolute volatile emissions (taken as the sum of the concentration of individual volatile compounds) (Fig. 1) and relative percentages of different VOC classes from undamaged (Fig. 2A) and mechanically damaged plants (Fig. 2B) were similar. However, VOCs, collected after the 8 hr initial caterpillar damage, varied significantly compared to the other two groups, and increased almost linearly over time, until sampling days 2–4 when the VOCs decreased sharply (Fig. 1). Specifically, in the VOC profile of 1-day herbivore-damaged plants, the release of two classes of VOCs, green leaf volatiles (GLVs) and terpenoids (we named them increased group, IG), were obviously increased, which made up the bulk of VOCs after the initial 8 hr of caterpillar damage (Fig. 2C). The increase in 1-day VOC concentration could be attributed to 6 GLVs [3-hexanol, (*Z*)-3-hexen-1-ol, (*E*)-2-hexen-1-ol, (*Z*)-3-hexenyl acetate, 2-hexen-1-ol, acetate, and 3-hexanone] and 6 terpenoids [β-myrcene, (*E*)-β-ocimene, (*Z*)-β-ocimene, linalool, caryophyllene, and α-farnesene] (Table 1). Of these, (*Z*)-β-ocimene (F = 228.340, DF = 5, P < 0.001) and 2 GLVs [(*Z*)-3-hexen-1-ol (F = 522.61, DF = 5, P < 0.001) and (*Z*)-3-hexenyl acetate (F = 57.25, DF = 5, P < 0.001)] were predominant in all treatments, and showed significantly higher amounts after the initial 8 hr of caterpillar damage (treatment 3) compared to the other treatments (Table 1). The absolute volatile emissions of hydrocarbons also increased after herbivory initial damage. However, the other five classes of VOCs, alcohols, aldehydes, ketones, esters, and benzenoids (we named as decreased group, DG), decreased after the initial 8 hr of caterpillar damage (Fig. 2C). Of these, total emissions of 2 aldehydes [nonanal and decanal] and 1 ketone [6-methyl-5-hepten-2-one] significantly decreased (F = 23.62, DF = 5, P < 0.001 for nonanal; F = 225.21, DF = 5, P < 0.001 for decanal; F = 26.66, DF = 5, P < 0.001 for 6-methyl-5-hepten-2-one) (Table 1).

In contrast to the 1-day group, in the VOC profiles of 2- to 3-day herbivore-damaged plants, the increased groups (IG) decreased or disappeared (Fig. 2D,E). Of these, several compounds obviously decreased, including 3 GLVs [(*Z*)-3-hexen-1-ol, (*E*)-2-hexen-1-ol, and (*Z*)-3-hexenyl acetate (F = 522.61, 640.41, 57.25, respectively, DF = 5, P < 0.001)] and 4 terpenoids [(*E)*-β-ocimene, (Z)-β-ocimene, linalool, and α-farnesene (F = 64.12, 228.34, 533.58, 253.00, respectively, DF = 5, P < 0.001)] (Table 1). However, the decreased groups (DG) increased. Specifically, 1 alcohol [2-decen-1-ol], 3 aldehydes [octanal, nonanal, and decanal], and 1 ketone [6-methyl-5-hepten-2-one] obviously increased (Table 1).

The variation trend was further developed on the fourth day after the initial herbivore-damage (treatment 6), when extremely low amounts of increased groups (IG) (GLVs and terpenoids) were detected, accompanied with higher amounts of decreased groups (DG) (alcohols, aldehydes, ketones, esters, and benzenoids) (Table 1, Fig. 2F).

### Identification of plant volatiles qualitatively

**Figure 3:**
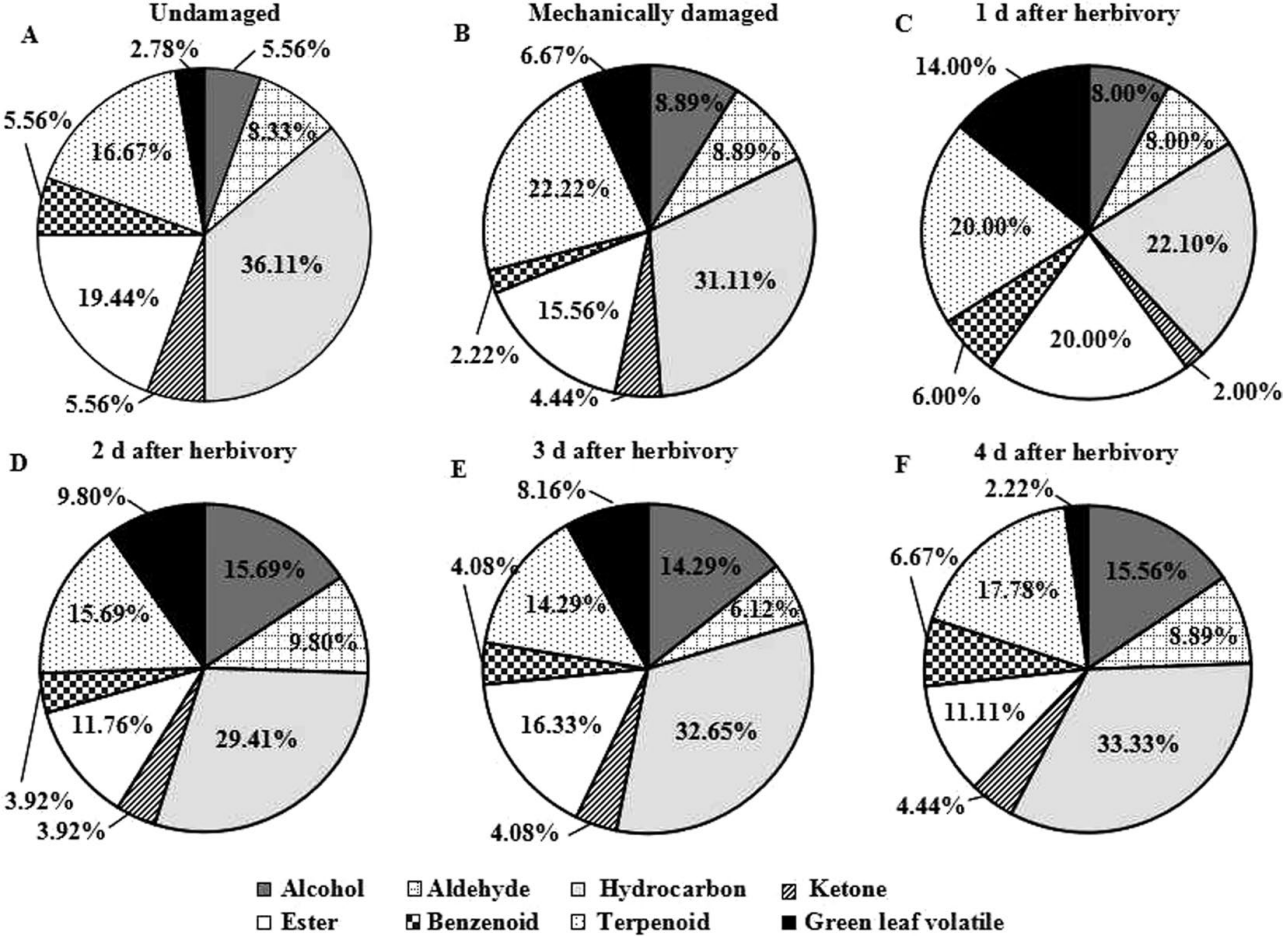
Qualitative proportions of major classes of volatile organic compounds in the headspace collected from undamaged, mechanically damaged, and herbivore-damaged *Aquilaria sinensis* plants damaged by leaf-feeding larvae of *Heortia vitessoides* over a 4-d period.

The absolute numbers (Fig. 1) and relative percentages of different VOC classes between undamaged (Fig. 3A) and mechanically damaged plants (Fig. 3B) were similar. However, the number of VOCs gradually increased in the herbivore-damaged plants (Fig. 1). Specifically, for VOC profiles of 1-day herbivore-damaged plants, the number of GLVs, terpenoids, and alcohols increased after the initial 8 hr of caterpillar damage. In comparison, the other VOC classes varied to a lesser degree after the initial caterpillar attack (Fig. 3C). Of the VOCs with increased variations, 6 GLVs [3-hexanol, (*Z*)-3-hexen-1-ol, (*E*)-2-hexen-1-ol, 3-hexanone, (*Z*)-3-hexenyl acetate, and 2-hexen-1-ol, acetate], 4 terpenoids [β-myrcene, linalool, epiglobulol, and caryophyllene], 3 alcohols [3-methyl-4-heptanol, 1-octen-3-ol, and 2-pentadecyn-1-ol] and 5 esters [butyl isobutyrate, heptyl butanoate, hexyl acetate, methyl benzoate, and ethyl hexadecanoate] emerged after the initial caterpillar attack, which were completely absent in undamaged plants. In contrast, 1 alcohol [2-decen-1-ol], and 1 aldehyde [octanal] disappeared after the initial 8 hr of caterpillar damage (Table 1).

Those VOCs which emerged during the initial damage disappeared; in particular, GLVs gradually decreased or disappeared during the subsequent 2- to 4- sampling dates (Fig. 3D,E,F). Of these, 4 GLVs [2-ethyl-1-hexanol, 3-hexanol, 3-hexanone, and 2-hexen-1-ol, acetate] and 2 terpenoids [β-myrcene, caryophyllene] disappeared in 2- to 4-day herbivore-damaged plants compared with the VOC profile of 1-day herbivore-damaged plants. However, 1 alcohol [2-decen-1-ol], and 1 aldehyde [octanal] came back during the subsequent sampling dates (Table 1). VOC profiles of 4-day herbivore-damaged plants (Fig. 3F) were almost similar to undamaged plants qualitatively (Fig. 3A).

### PCA and hierarchical cluster analysis

**Figure 4:**
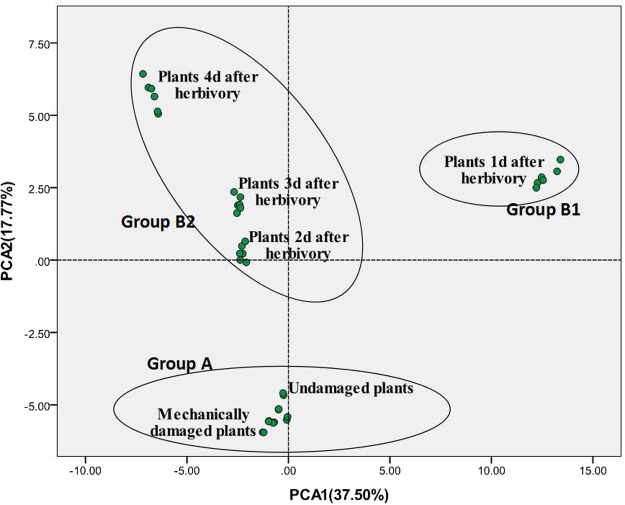
Scores plot of the principal component analysis (PCA) of headspace volatiles from undamaged, mechanically damaged, and herbivore-damaged *Aquilaria sinensis* plants damaged by leaf-feeding larvae of *Heortia vitessoides* over a 4-d period. Each single symbol (green circles) represents a sample. N = 6 for each treatment group. Black circles represent classification of these plants. The x-axis represents the first principal component (PC-1) and the y-axis represents the second principal component (PC-2), which accounted for 37.50% and 17.77% of the total variation, respectively.

Principal component analysis (PCA) clearly segregated the overall composition of the headspace volatile blends collected from the six plant treatments (Fig. 4). A scatter plot of the first and second principal components showed that principal component 1 was more discriminating than principal component 2. The two principal component axes accounted for 55.27% of the total variation in VOCs. The first PCA accounted for 37.50% and the second PCA accounted for 17.77%.

PCA also segregated the volatile blends into three groups according to the behavioural effects on *H. vitessoides* females. Group A, comprised of the volatile blends detected in the undamaged and mechanically damaged *A. sinensis* plants, was the volatile blend that attracted female moths. Non-attractive blends clustered in two distinct groups: Group B1 was a volatile blend from 1 d after herbivory plant damage and Group B2 was comprised of blends from 2 d, 3 d, and 4 d after herbivory damage of *A. sinensis* plants. The volatile blend from 1d after herbivory damaged plants (Group B1), although unattractive, occupied a different position in the PCAs compared to the other three unattractive blends (Group B2) (Fig. 4). The number of compounds detected in this unattractive blend (Group B1) was exceptionally high, especially the terpenoids and green leaf volatiles (Table 1).

**Figure 5:**
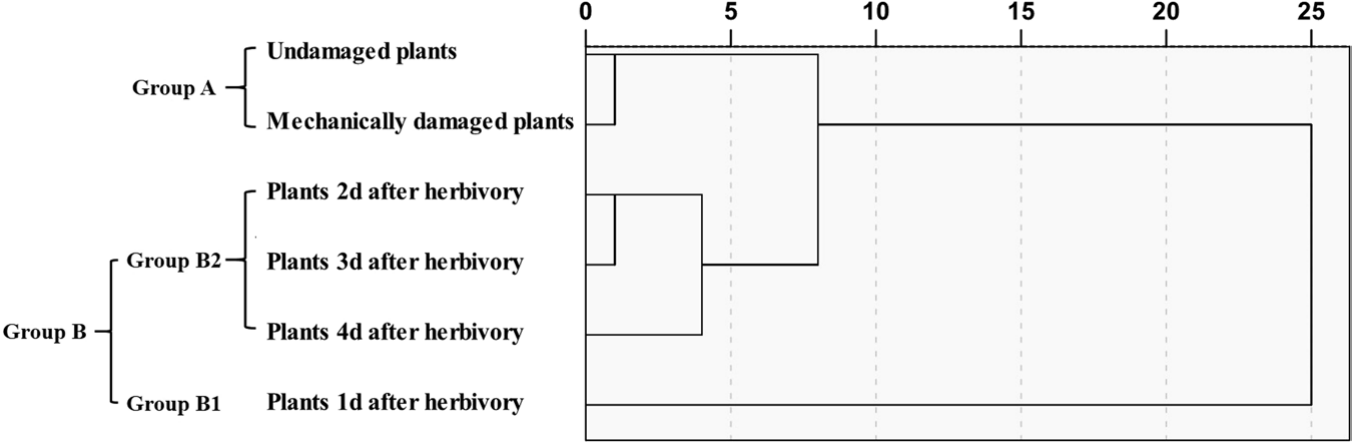
Phylogenetic tree of VOCs for the six treatments of *Aquilaria sinensis* plants from undamaged, mechanically damaged, and herbivore-damaged plants damaged by leaf-feeding larvae of *Heortia vitessoides* over a 4-d period.

Hierarchical cluster analysis between-groups linkage was used to analyse the volatiles derived from six treatments, at a distance >5 and <20. They were divided into three clusters (Group A, B1, B2, Fig. 5). System clustering results were consistent with the PCA results.

### Antennal responses of *H. vitessoides* to plant volatiles

**Figure 6:**
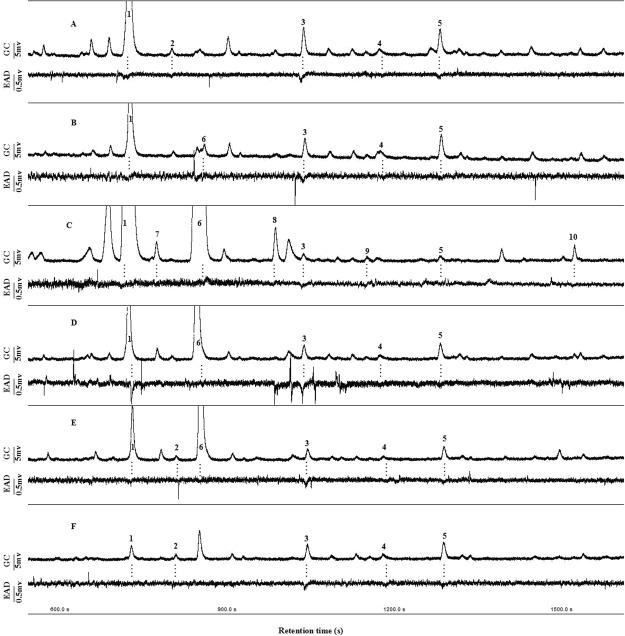
Simultaneously recorded GC-EAD using the antennae of *Heortia vitessoides* females in response to volatiles collected from undamaged (**A**), mechanically damaged (B), and herbivore (*H. vitessoides*) damaged *Aquilaria sinensis* plants 1 d (**C**), 2 d (**D**), 3 d (**E**), and 4 d (**F**) following insect attack. The upper trace represents the Flame Ionization Detector (FID) response and the lower trace represents the female-consistent EAD response. The EAD-active compounds were as follows: (1) (*Z*)-β-ocimene; (2) octanal; (3) nonanal; (4) 2-decen-1-ol; (5) decanal; (6) (*Z*)-3-hexenyl acetate; (7) hexyl acetate; (8) (*Z*)-3-hexen-1-ol; (9) 1-octen-3-ol; and (10) methyl benzoate.

In total, 10 compounds from the headspace of *A. sinensis* plants elicited antennal responses from *H. vitessoides* females: (1) (*Z*)-β-ocimene; (2) octanal; (3) nonanal; (4) 2-decen-1-ol; (5) decanal; (6) (*Z*)-3-hexenyl acetate; (7) hexyl acetate; (8) (*Z*)-3-hexen-1-ol; (9) 1-octen-3-ol; and (10) methyl benzoate. Of these, the terpenoid [(*Z*)-β-ocimene (peak 1)] and aldehydes [nonanal (peak 3) and decanal (peak 5)], common compounds among the six treatments, elicited consistent antennal responses (Fig. 6).

Five compounds from the undamaged plants (treatment 1), including (*Z*)-β-ocimene (peak 1), octanal (peak 2), nonanal (peak 3), 2-decen-1-ol (peak 4), and decanal (peak 5), elicited antennal responses (Fig. 6A). Five compounds from mechanically damaged plants (treatment 2) elicited antennal responses; (*Z*)-3-hexenyl acetate (peak 6) was characteristic, while octanal (peak 2) was not characteristic of electroantennographic detection (EAD)-active compounds from mechanically damaged plants, compared to undamaged plants (Fig. 6B).

Compared to undamaged plants, both octanal (peak 2) and 2-decen-1-ol (peak 4) were not present in volatiles collected after herbivory initial damage (Fig. 6C). In addition, the other five characteristic VOCs, which emerged after the initial 8 hr of caterpillar damage and made up the bulk of VOCs in the sample date, also elicited antennal responses. These five VOCs included 2 GLVs [(*Z*)-3-hexenyl acetate (peak 6) and (*Z*)-3-hexen-1-ol (peak 8)], 1 ester [hexyl acetate (peak 7)], 1 alcohol [1-octen-3-ol (peak 9)], and 1 benzenoid [methyl benzoate (peak 10)] (Fig. 6C).

EAD-active profiles of *A. sinensis* plants on the second, third, and fourth day after the initial 8 hr herbivore-damage were similar (treatment 4–6) (Fig. 6D–F). The common compounds, (*Z*)-β-ocimene (peak 1), nonanal (peak 3), 2-decen-1-ol (peak 4), and decanal (peak 5), elicited consistent antennal responses. The five compounds, which emerged after the initial 8 hr damage, disappeared gradually. In particular, the EAD-active odour profiles of plants on the fourth day after the initial 8 hr herbivore-damage (treatment 6) were completely similar to undamaged plants (treatment 1) (Fig. 6A,F).

In summary, the antennae of *H. vitessoides* females responded to not only the most abundant compounds, such as (*Z*)-β-ocimene (peak 1), (*Z*)-3-hexenyl acetate (peak 6), and (*Z*)-3-hexen-1-ol (peak 8), but also to the less abundant compounds, such as octanal (peak 2), nonanal (peak 3), 2-decen-1-ol (peak 4), decanal (peak 5), hexyl acetate (peak 7), 1-octen-3-ol (peak 9), and methyl benzoate (peak 10) in all treatments (Fig. 6).

### Wind tunnel bioassays

**Figure 7:**
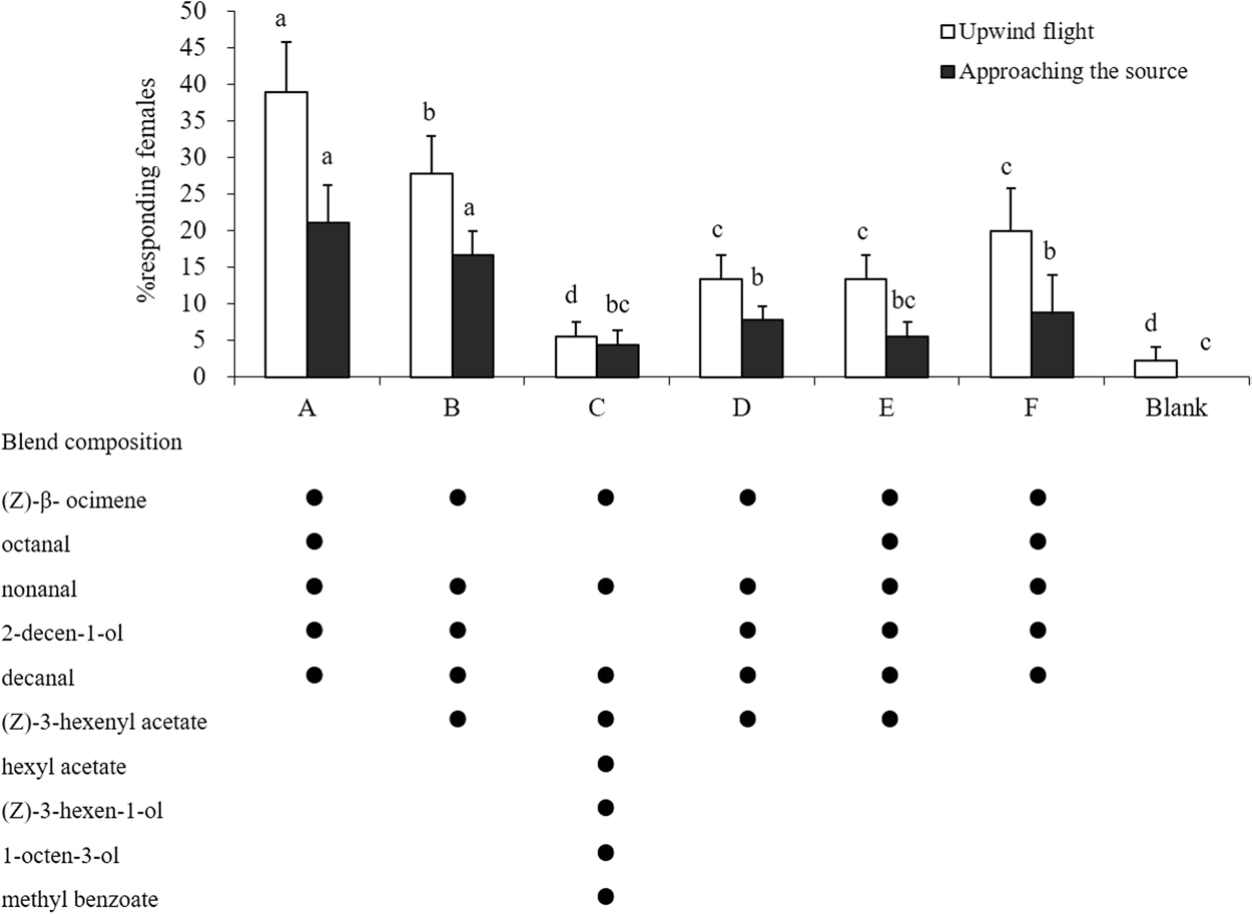
Number of responses of mated *Heortia vitessoides* females to synthetic blends from undamaged (**A**), mechanically damaged (**B**), herbivore (*H. vitessoides*) damaged *Aquilaria sinensis* plants 1 d (**C**), 2 d (**D**), 3 d (**E**), 4 d (**F**) following insect attack, and blank in the wind tunnel. Synthetic blends from different plant treatments eliciting consistent antennal responses in female *H. vitessoides* were prepared according to the natural ratios of each compound to the headspace collections (Table 2). Females were scored for upwind flights over 120 cm (white columns) and for approaching the source within 5 cm (black columns). Bars with the same colour and different letters were significantly different.

All six EAD-active blends that were tested stimulated *H. vitessoides* female upwind flights and approaches to within 5 cm of the source (Fig. 7). Synthetic blends mimicking undamaged plant VOCs (A) had the strongest attraction to females in the wind tunnel; 38.89% of females flew over 120 cm upwind, and 21.11% arrived within 5 cm of the source. The number of female upwind flights elicited by synthetic blend A was significantly higher than flights elicited by the other blends (F = 25.900, DF = 6, P < 0.001). Blend B, containing five compounds identified in the headspace of mechanically damaged plants, was the second most attractive to females. This blend resulted in 27.78% of females flying upwind and 16.67% approaching the source. Compared with blend A (mimicking undamaged plant VOCs) and blend B (containing five compounds identified in the headspace of mechanically damaged plants), the synthetic blends containing compounds released from herbivore-damaged *A. sinensis* plants (C, D, E, and F) elicited significantly fewer females upwind flights and landings near the source (F = 25.900, DF = 6, P < 0.001 for upwind flights; F = 15.127, DF = 6, P < 0.001 for landings near the source). There were no differences in the number of female upwind flights or landings near the source among the 4 blends from herbivore-damage plants (C, D, E, and F). The control (hexane solvent) did not induce any females to land near the source.

### Y-tube bioassays

**Figure 8:**
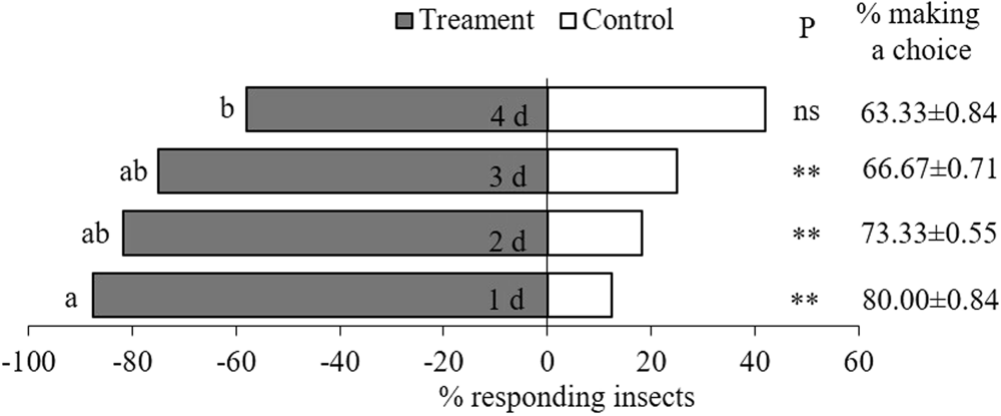
Olfaction selection preference and responsiveness (i.e. percentage of females making a choice) of predatory *Cantheconidea concinna* to different treatments of *Aquilaria sinensis* plants in a Y-tube olfactometer. Undamaged plants were used as control. Treatment groups consisted of 1 d, 2 d, 3 d and 4 d mean herbivore (*Heortia vitessoides*) damaged *A. sinensis* plants. *P*-values are based on chi-square test: ^**^P < 0.001, ns = *P* ≥ 0.05. Different lower-case letters on the left side of the bar indicate significant differences (one-way ANOVA followed by least significant difference’s multiple comparison test, *P* < 0.05).

In the dual-choice bioassay, *C. concinna*, a predator of *H. vitessoides* larvae, preferred VOCs from herbivore-damaged plants to those from undamaged plants (Fig. 8). The predator was attracted by the odour of 1-day herbivore-damaged plants (X^2^ = 111.386, N = 30, P < 0.001), 2-day herbivore-damaged plants (X^2^ = 81.820, N = 30, P < 0.001), and 3-day herbivore-damaged plants (X^2^ = 50.000, N = 30, P < 0.001). The effect of 4-day herbivore-damaged plants odour was not significant (X^2^ = 5.120, N = 30, P = 0.034). Results from the one-way ANOVA showed that olfactory response rates of *C. concinna* to the odours from *A. sinensis* plants differed among damage treatments (F = 4.04, P < 0.05). *C. concinna* adults showed a preference for recently herbivore-damaged plants (1–3 d of herbivore damage) over plants with 4 d of damage.

## Discussion

Plants commonly respond to herbivore attacks by releasing HIPVs^6,20^. The production and release of HIPVs can directly and indirectly affect herbivore performance and mediate interactions with other community members. Thus, HIPVs act as signals to herbivores, their natural enemies, and neighbouring plants^6,8^. These components have been well described in agricultural ecological systems, such as maize^17^, rape, and cotton^21,22^. Herein, we studied a tritrophic system which includes the herbivore *H. vitessoides*, its host tree *A. sinensis*, and its predator *C. concinna*. Our studies indicate that previously described tritrophic interactions in agricultural crop systems also apply to a forest ecosystem.

HIPVs may discourage oviposition of herbivores on damaged plants and may, therefore, be beneficial in reducing herbivore density^23^. Our behavioural bioassays showed that mated *H. vitessoides* females preferred VOC blends mimicking healthy, undamaged *A. sinensis* plants to those containing VOCs emitted by herbivore-damaged plants. This suggests that *H. vitessoides* females detect and assess VOCs released by *A. sinensis* to locate suitable oviposition sites and avoid the unsuitable sites. Similarly, in a dual-choice test weevils have been shown to prefer undamaged clover leaves to weevil-damaged leaves^24^. In behavioural bioassays, alate *Aphis gossypii* preferred the odour from undamaged cotton seedlings to that from *A. gossypii*-infested plants^25^. Recognition and the ability to locate suitable host plants using plant volatiles is beneficial for the offspring of insect herbivores^20^. Female insects generally prefer healthy, intact plants as oviposition sites, as these are more likely to provide newly hatched larvae with enough food resources. Thus, this strategy reduces the strength of intraspecific food competition, and increases individual survival and population growth^26^. In a previous study, we showed that mated *H. vitessoides* females were more attracted to young leaves than to old leaves of *A. sinensis* plants, and suggest that the former provides suitably tender food for freshly hatched, delicate young larvae^12^.

Many studies have shown that HIPVs may act as an important signal for natural enemies to locate their host/prey^27,28^. HIPVs are likely to act as important cues for natural enemies to locate damaged plants, and by extension, the herbivores attacking those plants, and, thus, may act as indirect plant defences^23,29^. Females laying eggs on undamaged plants also reduce the risk of parasitoid and predator attacks on larvae^29^. HIPVs are highly detectable and variable, and parasitoids and predators can distinguish these compounds to infer host suitability and even detect if hosts are parasitized or not^6^. HIPVs can increase predation and parasitism rates of herbivores, and, thus, reduce plant damage and increase reproductive output^20^. Our study demonstrates that *C. concinna* prefers the odours of herbivore-damaged plants to those from undamaged plants. Thus, HIPVs could be beneficial in attracting the predator *C. concinna* in response to insect attack.

Plants can produce various complex VOCs, which together make up the particular volatile spectrum of each species^29,30^. Some volatile compounds are continuously emitted, while many others are only released when plants are attacked by herbivores or mechanically damaged^7^. This damage response can play a key role in mediating multitrophic plant-insect interactions^8,31^. These complex volatile compounds resulting from damage tend to be released in greater variety and quantities than those from intact, healthy plants^6,9^. Numerous studies have shown both large quantitative increases and qualitative changes in VOC emissions as a result of mechanical and herbivore damage^20,21^. In particular, obvious differences were found between VOCs from herbivore-damaged *A. sinensis* plants compared to undamaged or mechanically damaged plants; herbivore damage elicited the release of a greater variety of VOCs, and in far greater quantities than mechanical damage. Herein, using mechanically-damaged plants as one of the treatments was valuable and allowed us to compare the difference between herbivore feeding and mechanical wounding to characterize a set of special HIPVs.

In general, the majority of HIPVs belong to green leaf volatiles (GLVs-C_6_ aldehydes, alcohols, and their esters), terpenoids, aromatics, and amino acid volatile derivatives^32^. Similarly, these classes detected in our study showed an increased emission pattern in response to herbivore feeding, most of which belonged to the increased groups (IG). However, HIPVs varied considerably over time in response to damage^33^. Different chemical classes have different change rhythms. Some GLVs are produced immediately after initial damage by the larvae of herbivores; the production and emission of these GLVs occurs almost instantaneously during the initial 1–2 hours after herbivore damage. While other chemicals, such as terpenoids, are released several hours after herbivore damage or the following day^21,34^; these HIPVs are synthetized *de novo* and emitted later^33^. Some studies have shown that there are different biosynthesis pathways, including autolytic oxidative breakdown of membrane fatty acids or nonmevalonate for different chemical classes.

All of the quantitative and qualitative changes in VOC emissions are always short-lasting. Once the damage ceases, the emission of these VOCs drops rapidly, making this a highly dynamic process^21,22,35^. In our study, the quantity and diversity of several HIPV classes, including GLVs or terpenoids, declined gradually soon after the attacks on the plant stopped. Simultaneously, several other classes, including aldehyde, alcohol, and ketone, almost disappeared after the initial damage, and recovered again during the subsequent 2–4 day sampling dates.

The rhythm of these changes in different chemical classes is consistent with the behaviour results in the wind tunnel. The increase in GLVs [(*Z*)-3-hexenyl acetate, (*Z*)-3-hexen-1-ol] and terpenoids [(*Z*)-β-ocimene] and the decrease in aldehydes [octanal, nonanal, and decanal] and alcohols [2-decen-1-ol] during the initial damage correspond to less attraction of *H. vitessoides* females to the synthetic blends that mimic the initial damaged plant VOCs. Conversely, the decrease of GLVs and terpenoids and the recovery of aldehydes and alcohols during the 2–4 day sampling dates correlated with the recovered attraction of females in the wind tunnel for synthetic blends that mimic the 2–4 day damaged plants. These data imply that HIPVs, including GLVs and terpenoids, are repellent for female *H. vitessoides*. However, some aldehydes and alcohols are attractive to female *H. vitessoides*. Most studies have found that adult moths are repelled by host volatiles released by conspecific larval feeding^16,17,36^, although this is by no means universal^37^. Clearly, more behavioural studies are needed to assess the impact of these various volatile components on insect behavior^38^.

Behavioural responses to VOCs from plants do not always mirror electrophysiology results in some insects. Not all GC-EAD active components are attractive in the behaviour assay and may act as a repellent. Wee *et al*.^24^ reported that lemon leaf volatiles elicited electrophysiological responses in weevils, but weevils were repelled by these compounds in behavioural bioassays. In our study, the three GC-EAD components [(*Z*)-3-hexenyl acetate, (*Z*)-3-hexen-1-ol, and (*Z*)-β-ocimene] were repellent in the wind tunnel assay. The amplitude of EAD is also not consistent to its behavioural activity. In our study, mated *H. vitessoides* females responded consistently and strongly to VOC blends mimicking headspace collections from herbivore-damaged *A. sinensis* plants in electroantennographic tests, but behavioural responses to these compounds in wind tunnel bioassays were weaker. Some studies have found discrepancies between electrophysiological and behavioural responses to VOCs in herbivores^24,39^. For example, the highest EAD responses from *Pandemis heparana* moths were obtained with the terpenes, linalool and DMNT, which are often key volatiles in herbivore deterrence^36,40^. The addition of the four compounds that elicited the smallest antennal responses resulted in improved levels of upwind flight of female grape berry moths (*Paralobesia viteana*)^41^.

## Methods

### Insects

*H. vitessoides* eggs were provided by Huazhou Green Life Co. Ltd (Guangdong, China) from the *A. sinensis* fields in the Chinese Medicinal Material Production Base (CMMPB). Newly hatched larvae were mass-reared for three instars in glass containers (diameter: 20 cm, height: 30 cm), and then separately transferred to smaller glass containers (diameter: 3 cm, height: 10 cm) with fresh *A. sinensis* leaves. Adults were provided with a 10% sugar-water solution on water-soaked cotton. All insects were reared in a climate-controlled room (25 ± 2 °C, 70 ± 5% RH, L16:D8).

Late-third to fourth-instar *H. vitessoides* larvae were used in the tests to induce the HIPVs. Larvae were starved overnight prior to all experiments to encourage active feeding immediately after being placed on plants. In behavioural assays, mated females were used. To obtain mated females, the newly emerged adult couples were placed in a cage (200 × 200 × 200 cm) with *A. sinensis* at a 2:1 ratio of male:female to ensure mating. Only females laying eggs were used in the wind tunnel bioassays. None of the females used in tests had previously been exposed to any of the tested odours and each was used only once^42^.

*C. concinna* nymphs, the primary predator of *H. vitessoides* larvae, were collected from the same fields as the original *H. vitessoides* eggs and reared in smaller glass containers (diameter: 3 cm, height: 10 cm) under the same conditions as their hosts. Late-third to fourth-instar *H. vitessoides* larvae were provided as food to *C. concinna* nymphs and adults. *C. concinna* adults used in Y-tube trials were 1–2 d old. All adults were starved overnight prior to trials and none had been exposed to any host plant or prey odour.

### Plant materials and treatments

Healthy potted *A. sinensis* seedlings were cultivated in thin-meshed gauze cages (200 × 200 × 200 cm). No *H. vitessoides* damage occurred during cultivation and no insecticides or other specific treatments against *H. vitessoides* were used at the study site during the trials. The environmental condition in the thin-meshed gauze cages was set at a 16 hr light/8 hr dark photoperiod.

The tested *A. sinensis* seedlings were from the thin-meshed gauze cages. Plants that were about 70 cm tall were used in all experiments. Plants were individually wrapped in gauze mesh during the whole trials. These plants were randomly divided into three groups, including undamaged plants (treatment 1), plants cut using a razor (treatment 2), and plants infested with *H. vitessoides* larvae (treatment 3, 4, 5, 6). Throughout the experiment, all treatments were kept separate, to prevent possible plant-to-plant transmission of airborne signals.

Seven treatments were administered as follows. Treatment 1 (undamaged plants)-plants were enclosed by a fine-mesh gauze with enough space between the plant and gauze to protect from any insect herbivory and damage during the experiments. Treatment 2 (fresh mechanically damaged plants)-100 cuts (1 cm in length) were made on the leaves of each plant with a razor blade to simulate the damage caused by late-third to four-instar *H. vitessoides* larvae. Treatments 3–6 (*H. vitessoides* damaged plants)-100 late-third to four-instar *H. vitessoides* larvae were placed on each test plant and allowed to feed on the plant. Treatment 7 (clean bags)-odour samples were collected from clean roasting bags (40.6 × 44.4 cm; Reynolds roasting bag, Richmond, Virginia, USA) as a control.

For mechanical-damage treatment 2, the plants were immediately placed inside the volatile collection system (see below) after mechanical damage. Collections were performed for 8 h. For the herbivory treatments 3–6, each entire plant was individually wrapped in gauze mesh to prevent the larvae from escaping during the experiment. After 8 h of feeding, the gauze mesh and larvae were removed from the infested plant. Volatile collection experiments began after the removal of the larvae. Collections were conducted for 8 h every day for a successive 4 d period, corresponding to the four treatments, 3–6. Each collection was made at the same time (20:00–04:00) each day, corresponding to the oviposition peak period of *H. vitessoides*. The plants were weighed immediately after collection. Each treatment was repeated six times, with different plants, cut damage, and test larvae.

### Plant volatile collections

We used a headspace collection system to collect headspace volatiles from plants. Living test plants were placed in a clean roasting bag. The bag was sealed around the plant stem with a self-sealing strip about 20 cm above soil-height^34^. Humidified, charcoal-filtered air was pulled through the bag with a pump (Beijing Institute of Labour Instruments, China) at 300 ml·min^−1^ and passed over an adsorbent cartridge. The adsorbent cartridge was a 0.5 × 10 cm glass column containing 50 mg of adsorbent (80/100 mesh, Supelco, Bellefonte, PA, USA). The Porapak Q (50 mg, 80–100 mesh, Supelco, Bellefonte, PA, USA) was held between plugs of glass wool. Each sample was aerated for 8 h. Volatiles were eluted from the adsorbent cartridge with 500 μl redistilled hexane at room temperature. An internal standard of 0.5 μg of benzaldehyde (99%, Fluka Production) was added to the extract for chemical quantification^43^. The final extracts were reduced to 50 μl using a slow stream of nitrogen and then subjected to gas chromatography mass spectrometry (GC-MS) and gas chromatography-electroantennographic detection (GC-EAD). If not used immediately, extracts were stored in glass vials at −18 °C until use.

### GC-MS

Headspace extracts were analysed with an Agilent Technologies 6890 N gas chromatograph linked to a 5973 mass spectrometer (Palo Alto, CA, USA) with a polar DB-Wax or non-polar DB-5 fused silica column (both 30 m × 0.25 mm × 0.25 μm; J&W Scientific, Folsom, CA, USA). The column oven temperature was held at 50 °C for 1 min, raised to 120 °C at 3 °C·min^−1^, and then increased to 240 °C at 10 °C·min^−1^ for 10 min. Helium (1.0 ml·min^−1^) was used as the carrier gas. Splitless injection (2 μl) was used with an injector temperature of 250 °C. The transfer line was set at 280 °C. Compounds were identified based on comparison with the retention times and mass spectra of synthetic standards. Windows NT/MASS Spectral Search Program (Version 1.7) software was used for the data analysis^44,45^.

### GC-EAD

Headspace extracts (2 µl) were analysed using the Gas Chromatography-Electroantennographic Detection (GC-EAD) system: an Agilent Technologies 7890 N GC coupled with an electroantennogram detector (Syntech, Hilversum, The Netherlands). Column and oven temperature programs were as previously described for GC-MS. Injector and detector temperatures were 250 °C and 230 °C, respectively. Nitrogen was used as the carrier gas at a constant flow of 1.0 ml·min^−1^. The outlet of the GC column was split in a 1:2 ratio between the flame ionization detector (FID) and a cut mated *H. vitessoides* female antenna through a heated (280 °C) transfer line. The antenna was mounted in a holder with two metal electrodes using conductive gel (Spectra 360; Parker Laboratories, Fairfield, NJ, USA). The electrode was connected to a high impedance DC amplifier (IDAC-4; Syntech). Compounds eluting from the GC column were delivered to the mounted antenna through a glass tube (12 × 0.8 cm), carried by a humidified and purified supplemental airflow. The antennal signal and the FID signal were simultaneously recorded and analysed using Syntech software (Hilversum, The Netherlands). Each antenna used for the tests was cut from a different mated female and used only once. Each sample was tested three times.

### Chemicals

Nonanal (97%), decanal (97%), and octanal (98%) were obtained from Fluka Production (Buchs, Switzerland). (*Z*)-3-hexenyl acetate (97%) and (*Z*)-3-hexen-1-ol (98%) were bought from Carl Roth (Karlsruhe, Germany). Hexyl acetate (99%), methyl benzoate (99%), 2-decen-1-ol (97%), and 1-octen-3-ol (98%) were purchased from Sigma-Aldrich Co. (St. Louis, MO, USA). (*Z*)-β-ocimene (95%) was obtained from BOC Sciences (New York, USA). Compounds for which no standards were available were tentatively identified using the NIST-database^42^.

### Wind tunnel bioassays

Assessments of the attractiveness of synthetic chemical blends to mated *H. vitessoides* females were carried out in a plexiglas wind tunnel (flight section: 200 × 60 × 60 cm). Incoming air was filtered through activated charcoal and was blown by a horizontal fan at 0.3 m·s^−1^ at the point of release of the moths. The upwind and downwind ends of the tunnel were covered with gauze to prevent escape of moths^41,46^. All bioassays were performed from 20:00 to 04:00, which is the oviposition peak period of *H. vitessoides*^12^. During the tests, temperature and relative humidity of the wind tunnel were kept at 25 ± 2 °C and 75 ± 5%, respectively.

On the basis of the results of the GC-EAD analyses, VOCs from the six different treatments of *A. sinensis* eliciting antennal responses in the female of *H. vitessoides* were formulated in blends for the wind tunnel tests. Six blends of synthetic compounds were prepared in the ratios of GC-EAD-active VOCs as emitted by the corresponding treatments (Table 2). Chemicals were diluted with redistilled hexane (Sigma-Aldrich, St. Louis, MO, USA). For each blend, the formulations contained 0.5 mg of the most abundant compound and the others compounds were added in the same proportion as in the natural volatile mixture. Preliminary tests confirmed that these concentrations were adequate to elicit moth responses in the wind tunnel. The blends were released into the wind tunnel by means of a green rubber septum. A septum treated with hexane only and no scent lure served as a control^12,43,47^.

**Table 2:** Components and blend ratios for each blend used in the wind tunnel bioassays.

Compounds^a^	Amount loaded on rubber septum in six treatments^b^ (*μg*)					
	A	B	C	D	E	F
Alcohols						
2-decen-1-ol	10.33	31.04		7.73	6.11	71.89
(*Z*)-3-hexen-1-ol			7.88			
1-octen-3-ol			1.20			
Aldehydes						
octanal	8.74				6.11	75.12
nonanal	37.98	65.03	0.77	29.62	13.33	322.48
decanal	50.76	111.34	1.92	47.53	32.69	471.54
Esters						
(*Z*)-3-hexenyl acetate		32.45	46.69	5.07	1.92	
hexyl acetate			0.91			
methyl benzoate			4.28			
Terpenoids						
(*Z*)-β- ocimene	500.00	500.00	500.00	194.37	75.68	114.73

^a^In order of elution during gas chromatography.

^b^The six synthetic blends from undamaged (A), mechanically damaged (B) and herbivore-damaged *Aquilaria sinensis* plants at 1 d (C), 2 d (D), 3 d (E), 4 d (F) by *Heortia vitessoides* larvae.

Before the bioassays, the synthetic lures were loaded in individual rubber septa respectively. Each septum loaded with one of the test samples was placed in the centre of the upwind end of the tunnel (30 cm from upwind end), affixed to a holder, and used only once per day. In order to reduce the experimental error between different blends or same blends with different replications, all odour blends were deployed based on the same criteria. After each treatment, the flight section of the wind tunnel was washed with hexane, and then dried with an electric hair drier (HP 8200; Philips, Zhuhai, China)^43,48^.

One hour before the trial, all mated females were transferred to the wind tunnel room and allowed to acclimate to the conditions. Test females were introduced into the downwind end of the wind tunnel one by one. Three groups were run for each blend tested. The number of females tested in each group ranged from 25–30 depending on availability of mated females. Females were placed in a cylindrical gauze cage (diameter: 10 cm, height: 15 cm). The cylinder was closed with a solid lid on one side and placed in a holder at a height of 30 cm in the centre of the downwind end of the wind tunnel. At the beginning of the bioassays, the lid was removed, allowing the moths to leave the cage. Moth behaviour was scored as follows: (1) for >120 cm upwind oriented flight in the centre of the wind tunnel and (2) for coming within 5 cm of the odour source. The behaviour of each batch was observed for 20 min. Each female was used only once.

### Y-tube olfactometry

We tested the attraction of predatory *C. concinna* to damaged and undamaged plant tissue in a glass Y-tube olfactometer. Undamaged *A. sinensis* plants served as a control, and damaged plants were the same damage treatments described in ‘Plant materials and treatments’. The olfactometer consisted of two glass chambers (diameter: 10 cm) that were each connected with one of the two 20-cm-long arms of the olfactometer, and joined with a 20-cm-long common arm. The odour sources (potted plant tissue) were placed inside clean roasting bags, which were then connected to the extremities of each arm: one arm served as a control (undamaged plants) and the other held the test material (*H. vitessoides* damaged plants). Fine-meshed nylon gauze was inserted at the ends of the two arms of the Y-tube to prevent insects from reaching the plant tissue. Moistened, activated-charcoal-filtered air with the odour source was pumped into each arm at a flow rate of 300 ml·min^−1^. All bioassays were conducted during the photophase, which is the feeding period of *C. concinna*. Temperature and relative humidity in the Y-tube olfactometer were maintained at 25 ± 2 °C and 75 ± 5%, respectively.

One hour before the start of the bioassays, groups of insects were transferred to the Y-tube room and allowed to acclimatize in the observation room inside glass vials (15 × 3 cm). For the observations, insects were placed individually at the beginning of the common arm and observed for 10 min. Behaviour was recorded as choosing the test odour or the control if the insects entered the respective chamber. If the insects remained in the common arm of the Y-tube it was recorded that no choice had been made^49^. For each bioassay, 30 replicates were performed. Each insect was used only once in the bioassays. After each test, the Y-tube was washed with distilled water, acetone, and alcohol (v/v 90%), and then dried with an electric hair drier (HP 8200; Philips, Zhuhai, China).

### Data Analysis

Mean volatile concentrations in the headspace samples from different treatments, mean numbers of *H. vitessoides* females responding to each VOC blend in the wind tunnel, and percentage of *C. concinna* adults making each choice in Y-tube were each compared using one-way analysis of variance (ANOVA). Significant differences in the means were assessed using Tukey’s multiple range test (α = 0.01). Chi-square test was applied to analyse results from the Y-tube behavioural tests. To reduce the complexity of the multivariate VOC data, principal component analysis (PCA) was performed. PCA was applied to yield a 2D display of the multivariable data set and to graphically determine whether clustering of the six damage treatments (undamaged plants, mechanically damaged plants, and *H. vitessoides* damaged plants) occurred based on their overall VOC profiles^50^. All statistical analyses were performed using SPSS, version 16.0 (SPSS Inc. Chicago, IL, USA).
